# Mechanisms and Application of Bacteriophages in Regulating Intestinal Health of Livestock and Poultry

**DOI:** 10.1002/mbo3.70443

**Published:** 2026-10-21

**Authors:** Manman Song, Yuying Fan, Liguo Zhang, Wenhao Qi, Lin Li, Gang Zhao, Jianwei He

**Affiliations:** ^1^ School of Life Science Liaoning University Shenyang China; ^2^ College of Laboratory Animal Medicine Liaoning University of Traditional Chinese Medicine Shenyang China; ^3^ Liaoning Center for Animal Disease Prevention and Control Shenyang China; ^4^ College of Animal Science and Veterinary Medicine Shenyang Agricultural University Shenyang China

**Keywords:** antibiotic alternatives, gut microbiota, intestinal pathogens, phage cocktail, phage therapy

## Abstract

The escalating crisis of antibiotic resistance has driven the global livestock and poultry industry to seek effective alternatives. Bacteriophages (phages) are promising candidates because their high host specificity allows targeted elimination of key intestinal pathogens, including *Salmonella*, *Campylobacter*, pathogenic *Escherichia coli*, and *Clostridium perfringens*, while sparing commensal microbiota. This review summarizes the mechanisms by which phages promote intestinal health in livestock and poultry, covering pathogen lysis, modulation of gut microbiota, reinforcement of the intestinal mucosal barrier, and regulation of host immune responses. Evidence from broiler chickens, weaned piglets, and ruminants shows that phage therapy can improve growth performance and help control diseases such as necrotic enteritis and edema disease. We further discuss how administration route, dosage, timing, and formulation affect intestinal delivery, and how encapsulation systems such as microcapsules and liposomes improve phage survival under gastrointestinal conditions. Strategies to delay resistance evolution through phage cocktails, and combinations of phages with probiotics or plant extracts, are also evaluated, with a staged screening framework proposed for assessing compatibility prior to field deployment. Because positive results dominate the controlled‐trial literature, we critically appraise failed, limited, and non‐durable *in vivo* trials, attributing these outcomes to rapid clearance, anti‐phage immunity, inappropriate dosing, and spatial exclusion coupled with ecological mismatch. Commercial translation additionally depends on scalable production and downstream removal of endotoxins and host cell proteins at acceptable cost. Overcoming these challenges would allow phage‐based interventions to serve as precision antibiotic alternatives that support sustainable animal farming and global food security.

## Graphical abstract

Bacteriophages offer targeted, self‐limiting control of intestinal pathogens in livestock and poultry. This review integrates their biological properties, mechanisms of gut‐health promotion, key experimental evidence, application strategies, and current challenges, outlining a path toward sustainable animal production and global food security.

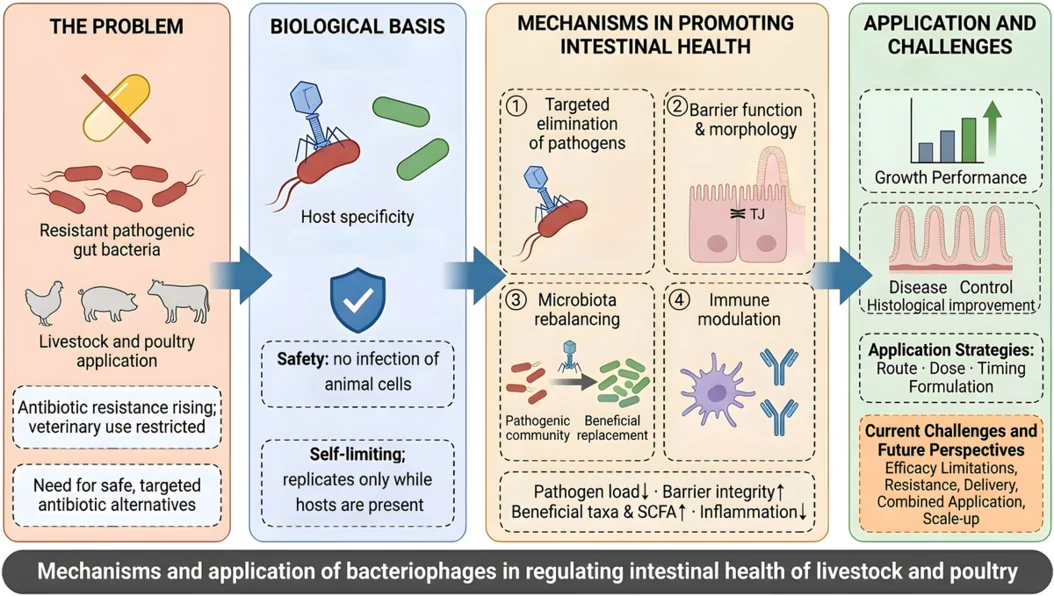

## 1 Introduction

Intensive, high‐density production systems have become the predominant model in livestock and poultry farming to meet the growing global demand for animal products. In these systems, antibiotics were long used as feed additives for disease prevention and growth promotion, contributing substantially to production efficiency over past decades. However, sustained subtherapeutic use exerts strong selective pressure and accelerates the emergence and transmission of antimicrobial resistance in food‐producing animals (Silva et al. 2023).

The animal gastrointestinal tract is a vast reservoir of resistance genes, and resistant bacteria can reach humans through fecal excretion, the food chain, and contaminated water (Vivarelli et al. 2026). The World Health Organization has identified antimicrobial resistance as one of the most critical public health threats of this century (Prestinaci et al. 2015), and many countries have banned or restricted antibiotic growth promoters in feed. Developing safe, effective, and sustainable alternatives has therefore become an urgent priority for the livestock and poultry industry (Rafiq et al. 2022).

Various antibiotic alternatives have been explored, including probiotics, prebiotics, organic acids, phytogenic compounds, and antimicrobial peptides. Most of these agents lack target specificity and cannot precisely eliminate defined pathogens, and the stability and cost‐effectiveness of some products at production scale remain to be validated. Alternatives capable of targeted pathogen control are therefore a central research direction in this field.

The gastrointestinal tract of livestock and poultry harbors a complex microbial ecosystem that interacts closely with the host epithelium and immune system. Its composition is dynamically shaped by animal age, genetic background, diet, rearing environment, and stress (Tardiolo et al. 2025). Notably, bacteriophages (phages) dominate the gut virome. Through continuous predation of bacteria, regulation of bacterial population dynamics, and mediation of horizontal gene transfer among bacteria, phages are profoundly involved in the dynamic regulation of the intestinal microecology and represent an endogenous force in maintaining microbial community homeostasis (Mahmud et al. 2024).

Under healthy conditions, beneficial bacteria occupy dominant ecological niches and suppress opportunistic pathogens, including *Salmonella* spp., *Escherichia coli* (*E. coli*), and *Clostridium perfringens*, through competitive exclusion, production of bacteriostatic substances, and modulation of mucosal immunity (Shi et al. 2022). Once this balance is disturbed, animals become susceptible to intestinal inflammation and production losses, and pathogens can further enter the food chain and threaten public health (Koyun et al. 2023). Therefore, achieving precise control of intestinal pathogens while preserving beneficial microbiota is a core task for sustainable animal production.

Phages, as a class of viruses capable of specifically infecting and lysing bacteria, hold considerable potential as antibiotic alternatives. Their high host specificity, self‐replication capacity, and relatively favorable safety profile suit them for intestinal health management in livestock and poultry. However, the practical application still faces multiple challenges, including bacterial phage resistance, variable delivery efficiency in the complex intestinal environment, and immature formulation and large‐scale production technologies.

This review summarizes the mechanisms of action, research progress, and application prospects of phages in regulating intestinal health of livestock and poultry. In this article, the biological characteristics of phages relevant to their applicability are described. We then analyze how phages protect intestinal health through targeted pathogen elimination, barrier reinforcement, microbiota modulation, and immune regulation, and evaluate key evidence from animal experiments. Finally, current challenges, formulation and combination strategies, commercial scalability, and future research directions are discussed, aiming to provide a reference for the scientific application and industrial translation of phage products in livestock and poultry farming.

## 2 Biological Characteristics of Bacteriophages and Their Applicability in Livestock and Poultry

### 2.1 Classification of Bacteriophages

Phages represent the most abundant and widely distributed biological entities on Earth, with an estimated total population exceeding 10^31^ particles, which is tenfold greater than the number of bacteria (Hatfull 2008). They are widely distributed across virtually all ecosystems, including soil, marine environments, freshwater, and the gastrointestinal tracts of humans and animals. Through long‐term coevolution with their bacterial hosts, phages constitute one of the largest biological components in the Earth's biosphere.

According to the latest taxonomic framework established by the International Committee on Taxonomy of Viruses (ICTV), phage classification has transitioned from traditional morphology‐based systems to genome‐based phylogenetic frameworks (Turner et al. 2023). In 2021, the ICTV formally abolished the three morphology‐based families (Myoviridae, Siphoviridae, and Podoviridae) and the order Caudovirales due to their polyphyletic nature and failure to accurately reflect evolutionary relationships, replacing them with the class Caudoviricetes, which encompasses all tailed phages with icosahedral capsids and double‐stranded DNA genomes based on shared evolutionary histories (Turner et al. 2021).

Beyond genomic classification, phages are further categorized into two major functional types, lytic phages and temperate phages, based on their interaction modes with host bacteria. This distinction is critical for candidate strain screening in phage therapy applications (Monteiro et al. 2019).

Lytic phages follow a strictly lytic cycle. The infection process initiates with the recognition and specific adsorption of receptor binding proteins (RBPs) located on the phage tail to specific receptors on the host bacterial surface, followed by injection of nucleic acid into the host cell (Nobrega et al. 2018). The phage then redirects host biosynthetic machinery to replicate its genome and produce structural proteins required for progeny assembly (Azam et al. 2021). After progeny virions are assembled, phage‐encoded holins permeabilize the bacterial cytoplasmic membrane, allowing endolysins to access and hydrolyze the peptidoglycan layer of the cell wall (Samir 2024). This process ultimately causes osmotic lysis and death of the host bacterium, releasing often tens to hundreds of progeny phages, a parameter known as the burst size, which can subsequently infect nearby susceptible bacteria and amplify the antibacterial effect (Elois et al. 2023). When the density of susceptible bacterial hosts falls below the threshold required to sustain replication, phage populations decline because of limited host availability, a feature referred to as the self‐limiting property of lytic phages. This self‐amplifying yet self‐limiting bactericidal mechanism makes strictly lytic phages particularly attractive candidates for phage therapy (Strathdee et al. 2023).

Temperate phages exhibit a more complex life cycle. Upon infecting the host, they do not immediately initiate the lytic cycle but instead integrate their genome into the host bacterial chromosome or replicate stably within the host cell as extrachromosomal plasmids, such as phage P1, existing as prophages that replicate synchronously with the host genome replication, a process termed the lysogenic cycle (Elois et al. 2023). Under specific inducing conditions, such as ultraviolet irradiation, chemical mutagens, or nutritional stress, prophages can be precisely excised from the host genome and re‐enter the lytic cycle to complete lytic replication.

In the application of phage therapy, strictly lytic phages are preferred, with temperate phages generally excluded, primarily for three reasons. First, temperate phages possess the genetic capability to integrate into the host genome and enter a dormant lysogenic state, thereby failing to guarantee immediate bactericidal activity and reducing therapeutic efficacy (Cook and Hynes 2025). Second, lysogenic conversion can confer new phenotypic characteristics to host bacteria, as temperate phages often serve as vectors for virulence genes, including those encoding exotoxins, or factors enhancing adhesion and biofilm formation, thereby potentially increasing pathogen virulence (Zhou et al. 2023). Third, temperate phages facilitate the dissemination of antibiotic resistance genes and other virulence factors through horizontal gene transfer mechanisms, particularly specialized transduction, although lytic phages must also be screened to exclude generalized transduction potential (Menouni et al. 2015; Pfeifer et al. 2022). Therefore, from perspectives of safety and efficacy, bacteriophage preparations intended for intestinal health management in livestock and poultry should primarily consist of rigorously characterized lytic phages, verified through genomic sequencing to ensure the absence of integrase genes, virulence factors, and antibiotic resistance markers, thereby ensuring precise bactericidal effects while minimizing biosafety risks (Pfeifer et al. 2022).

### 2.2 Host Specificity

Host specificity represents one of the core biological characteristics of phages and constitutes their fundamental advantage over broad‐spectrum antibiotics. As previously described, the precise molecular recognition between RBPs on the phage tail and specific receptors on the host bacterial surface determines the boundaries of the phage host range (Nobrega et al. 2018). These receptors encompass various bacterial surface structures, including lipopolysaccharides (LPS), outer membrane proteins (OMPs), pili, flagella, and capsular polysaccharides (Azam et al. 2021). Due to this receptor‐dependent recognition mechanism, most phages exhibit a narrow host range, often limited to specific species, serotypes, or even strains, although polyvalent phages with broader host ranges do exist in nature (Gambino et al. 2020).

The use of traditional broad‐spectrum antibiotics indiscriminately eliminates susceptible bacteria, including beneficial commensal flora indispensable to host health (such as *Lactobacillus* spp., *Bifidobacterium* spp., and *Faecalibacterium prausnitzii*), thereby leading to reduced gut microbiota diversity, compromised colonization resistance, and creating opportunities for secondary proliferation of resistant bacteria or opportunistic pathogens, ultimately resulting in intestinal dysbiosis and related diseases (Santacroce et al. 2023). In contrast, the precise targeting capability of phages enables specific elimination of target pathogens without disturbing the homeostasis of intestinal commensal flora. This precision approach helps maintain the structural and functional integrity of the gut microbiota, facilitating effective pathogen control while protecting the host's digestive, metabolic, and immune functions from unnecessary interference, thereby achieving precise and sustainable management of intestinal health in livestock and poultry (Gutiérrez and Domingo‐Calap 2020).

### 2.3 Safety Assessment

The safety of phages constitutes a fundamental prerequisite for their application in livestock and poultry production, necessitating systematic evaluation from multiple dimensions, including fundamental biological characteristics, in vivo application evidence, regulatory approval status, environmental impact, and potential risks.

From a fundamental biological perspective, bacteriophages, as obligate prokaryotic viruses, possess infection and replication mechanisms that are highly dependent on specific receptors on bacterial surfaces and intracellular replication machinery within bacterial cells (Azam et al. 2021). Since eukaryotic cells of livestock and poultry do not express these bacteria‐specific receptors, phages cannot complete their infection cycle or undergo autonomous replication within eukaryotic cells, constituting a fundamental safety foundation for bacteriophages (Sampath et al. 2025). Although studies have shown that phages can be internalized by eukaryotic cells and elicit immune responses through pattern recognition receptors, current evidence generally indicates that such interactions are safe for the host (Podlacha et al. 2024).

From the perspective of *in vivo* application evidence, extensive animal studies have demonstrated that rigorously screened lytic bacteriophage preparations do not exert significant toxic or adverse effects on animal growth performance, organ function, or intestinal commensal microbiota structure during the targeted elimination of intestinal pathogens (EFSA et al. 2023). Unlike antibiotics, phages are biological particles rather than chemical substances and do not generate persistent chemical residues in animal tissues or products. Following elimination of target bacteria, bacteriophage populations naturally decline due to the absence of available hosts for replication, thereby avoiding the sustained impact of antibiotic residues on food safety and the environment (Jiang et al. 2024).

From a regulatory perspective, bacteriophage applications have obtained preliminary approval in certain countries and regions. In the field of food safety, the U.S. Food and Drug Administration (FDA) and the U.S. Department of Agriculture (USDA) have approved several bacteriophage products targeting *Listeria monocytogenes* (such as ListShield [Perera et al. 2015] and Listex P100 [Rodríguez‐Melcón et al. 2018]) for pathogen control on the surfaces of ready‐to‐eat meats, poultry products, and dairy products. The European Food Safety Authority (EFSA) and the European Medicines Agency (EMA) have also recognized phages as a promising antimicrobial alternative for food‐producing animals, explicitly supporting further research and calling for the establishment of appropriate regulatory frameworks to promote their practical application in reducing antimicrobial use and combating bacterial resistance (EMA et al. 2017). However, dedicated regulatory frameworks for phages as veterinary feed additives or veterinary medicinal products remain incomplete globally, with considerable variation in approval standards among countries. Large‐scale preparation, quality control standards, and industrial registration pathways for such products still await systematic standardization, representing a significant practical obstacle constraining the widespread adoption of bacteriophage products in the livestock and poultry sector (Gigante and Atterbury 2019).

Regarding environmental impact, bacteriophages, as biodegradable natural entities, do not generate persistent chemical pollution when excreted via feces into the external environment and are generally considered ecologically safer than chemical antibiotics (Guo et al. 2024). However, as biologically active particles, phages can maintain activity for a certain period in soil and aquatic environments, and their potential to facilitate horizontal gene transfer among bacteria in environmental reservoirs remains under ongoing investigation (DiPietro et al. 2023). Given that no intervention is entirely risk‐free, the long‐term ecological effects of large‐scale bacteriophage application in livestock and poultry production warrant further evaluation.

## 3 Mechanisms of Phages in Promoting Intestinal Health in Livestock and Poultry

Phages promote intestinal health in livestock and poultry through four interconnected mechanisms: targeted elimination of pathogenic bacteria, enhancement of intestinal barrier function, modulation of gastrointestinal microbiota, and regulation of host immunity (Figure 1). These mechanisms synergistically maintain gut homeostasis and improve animal performance.

**Figure 1:**
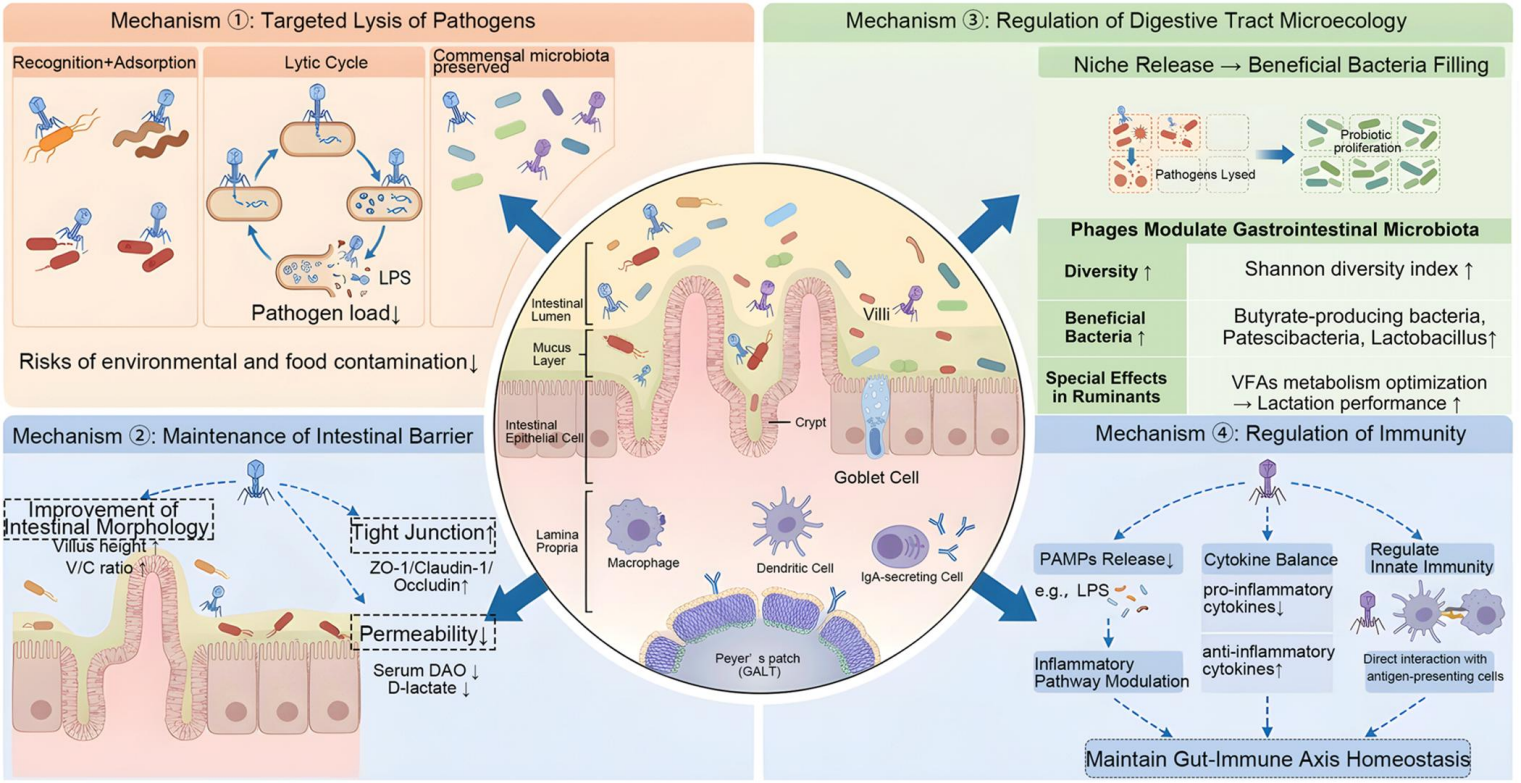
Multi‐dimensional mechanisms by which bacteriophages improve intestinal health in livestock and poultry. Bacteriophages enhance intestinal health through four interconnected mechanisms. Mechanism ①: Targeted Lysis of Pathogens. Phages recognize and adsorb to specific pathogens, initiating lytic cycles that reduce pathogen burden while preserving commensal microbiota, thereby lowering contamination risks. Mechanism ②: Maintenance of Intestinal Barrier. Phage intervention improves intestinal morphology, upregulates tight junction protein, and reduces permeability and inflammatory markers. Mechanism ③: Modulation of Gastrointestinal Microbiota. Pathogen lysis creates ecological niches subsequently colonized by beneficial bacteria, increasing microbial diversity and butyrate‐producing taxa. In ruminants, rumen microbiota remodeling optimizes volatile fatty acid (VFA) metabolism and lactation performance. Mechanism ④: Regulation of Immunity. Reduced pathogen‐associated molecular pattern attenuates inflammatory pathway activation, shifts the cytokine balance toward anti‐inflammatory profile, and enable direct phage interactions with antigen‐presenting cells, collectively maintaining gut‐immune axis homeostasis.

### 3.1 Targeted Elimination of Pathogenic Bacteria

Bacteriophages, as natural predators of bacteria, specifically lyse pathogenic bacteria in the intestine through the lytic cycle, which constitutes the fundamental prerequisite for their subsequent protective effects, including barrier protection, microbiota modulation, and immune regulation. In livestock and poultry production, various important intestinal pathogens, including *Salmonella*, *Campylobacter*, *E. coli*, and *Clostridium perfringens*, can be efficiently eliminated by specific bacteriophages (Żbikowska et al. 2020). This targeted bactericidal action not only effectively controls clinical infections but also reduces the risk of foodborne pathogen transmission to humans through the food chain at its source (Gigante and Atterbury 2019).

*Salmonella* is one of the most prevalent and serious foodborne pathogens in the livestock and poultry industry, capable of colonizing the intestinal tracts of various animals including poultry, swine, cattle, and sheep, causing enteritis, septicemia, and other diseases that severely threaten animal health and food safety (Zhang et al. 2022). Phages have demonstrated substantial application potential in controlling *Salmonella* infections. Studies have demonstrated that lytic phages screened against different serovars of *Salmonella* can efficiently eliminate host bacteria. Bacteriophage SM1 has been proven to effectively control *Salmonella Enteritidis* contamination in poultry bedding materials, reducing the risk of animal exposure and infection by decreasing pathogen loads in the environment (Rogovski et al. 2021). *In vivo* experiments in chicks have demonstrated that bacteriophage cocktails targeting *Salmonella Enteritidis* can reduce cecal colonization of the pathogen and regulate gut microbiota disorder, providing preliminary evidence for its biocontrol potential in poultry (Gao et al. 2026). In swine, bacteriophage cocktail therapy targeting intestinal *Salmonella* infections in weaned piglets has also effectively eliminated pathogens, with related improvements in intestinal health (Won et al. 2021). Collectively, these studies indicate that phages applied can directly lyse *Salmonella* in the intestine and environment, representing an effective strategy for controlling *Salmonella* infections.

*Campylobacter*, particularly *Campylobacter jejuni*, is one of the major foodborne pathogens causing human gastrointestinal infections, with transmission frequently associated with contaminated chicken meat (Henz et al. 2026). Due to high colonization levels and rapid transmission of *Campylobacter* in broilers, control during the farming stage has remained an industry challenge (Wales et al. 2019). Phages have shown unique application value in this field. A study isolated and characterized two broad‐host‐range phages CBP1 and CBP2 infecting both *Campylobacter jejuni* and *Campylobacter coli*, and confirmed their high infectivity against chicken‐derived isolates and significant ability to reduce *Campylobacter* loads on chicken meat (Ahn et al. 2026). These findings suggest that such phages have potential as biocontrol agents for reducing *Campylobacter* contamination in poultry production. Although some *Campylobacter* phages have relatively narrow host ranges, rationally formulated bacteriophage cocktails demonstrate significantly superior lytic efficacy compared to equivalent doses of single bacteriophages. A combined *in vitro* and *in vivo* study showed that combined application of *Campylobacter* phages yielded significantly better lytic efficacy than single phages or phage combinations from the same group, and markedly reduced intestinal *Campylobacter* loads in broiler models under near‐field conditions, demonstrating the prominent advantages of combined phage therapy in the control of *Campylobacter* in broilers (Peh et al. 2023). The core of this on‐farm intervention strategy lies in reducing pathogen colonization in poultry at the source, thereby interrupting the transmission chain to slaughter processing and final food products.

*E. coli* is an important opportunistic pathogen in the intestinal tract of livestock and poultry, characterized by diverse pathotypes (Abdullahi and Trabelsi 2025). Due to extensive antibiotic use, it has widely accumulated multidrug resistance, forming an important reservoir of resistance genes that can be transmitted to humans through the food chain, causing refractory infections (Silva et al. 2023). Studies have confirmed that lytic phages targeting pathogenic *E. coli* can cover the major prevalent serotypes in livestock and poultry farming, demonstrating good strain compatibility (Rashid et al. 2026). In poultry farming, novel lytic phages screened against avian pathogenic *E. coli* (APEC) can effectively reduce flock mortality and bacterial loads in animal infection models, providing reliable control measures for clinical prevention and control of multidrug‐resistant APEC infections (Jhandai et al. 2024). In swine farming, specific phages screened for mixed infections of intestinal pathogenic *E. coli* (IPEC) and extraintestinal pathogenic *E. coli* (ExPEC) can efficiently lyse hybrid pathogenic strains in mouse models (Imklin et al. 2024). A study has also evaluated bacteriophage cocktails against enterotoxigenic *E. coli* (ETEC F4LT1ST2) in weaned piglets, demonstrating reduced diarrhea burden and improved growth performance (Mahmud et al. 2026). In ruminant farming, lytic phages targeting multidrug‐resistant *E. coli* have been isolated from cattle, buffalo, and goat sources, demonstrating favorable genetic safety profiles and potential for veterinary application, although in vivo validation remains warranted (Anjay et al. 2026). These studies collectively demonstrate that *E. coli*‐specific phages exhibit favorable control efficacy across different animal hosts.

In addition to the three major pathogens mentioned above, the control potential of phages against anaerobic pathogens such as *Clostridium perfringens* (*C. perfringens*) has also received attention. *C. perfringens* is the primary pathogen causing necrotic enteritis in poultry, a disease that causes substantial economic losses in the global broiler industry (Waller et al. 2024). Against the backdrop of restricted use of antibiotic growth promoters, the incidence of necrotic enteritis has shown an increasing trend in some countries. Lytic phages against *C. perfringens* have been isolated and characterized from farming environments, and phage preparations can lyse a subset of pathogenic strains *in vitro*; *in vivo* trials confirm that it significantly alleviates intestinal lesions associated with necrotic enteritis in broilers (Keerqin et al. 2022). Nevertheless, these findings should be interpreted in the context of the highly structured intestinal environment in necrotic enteritis. Unlike broth‐based assays, where phages and planktonic bacterial cells are uniformly mixed, *C. perfringens* isolates from NE outbreaks exhibit strong binding to collagen, the major extracellular matrix component exposed upon mucosal damage (Sun et al. 2023), indicating lesion association rather than a freely planktonic lifestyle. Such lesion‐associated surfaces and potentially biofilm‐forming populations may reduce phage contact with *C. perfringens*, while the slow‐growing subpopulations within these structures may propagate phage poorly (Charlebois et al. 2020; Meneses et al. 2023). Thus, the in vivo performance of anti‐*C. perfringens* phages depends not only on host range and intrinsic lytic activity, but also on delivery, retention, penetration through mucosal and biofilm‐associated barriers, and access to susceptible bacterial receptors.

In summary, phages achieve targeted elimination of multiple key pathogens in the intestinal tract of livestock and poultry through their inherent lytic mechanisms. This direct bactericidal effect constitutes the fundamental prerequisite for subsequent bacteriophage‐mediated modulation of microbiota, protection of intestinal barrier integrity, and maintenance of immune homeostasis.

### 3.2 Enhancing Intestinal Barrier Function

A healthy intestinal barrier serves as the first line of defense against pathogen invasion, maintains intestinal homeostasis, and prevents systemic inflammatory responses. The intestinal barrier is a multi‐layered defense system consisting of physical, chemical, immune, and microbial components (Di Vincenzo et al. 2024). The core of the physical barrier consists of a monolayer of intestinal epithelial cells interconnected by tight junction (TJ) complexes composed of occludin, claudin family proteins, and zonula occludens‐1 (ZO‐1), which strictly regulate paracellular permeability (Pan et al. 2023). Once TJ integrity is compromised, increased intestinal permeability can trigger local inflammation and even systemic infections (Di Tommaso et al. 2021).

In livestock and poultry production, various intestinal pathogens compromise barrier integrity through distinct yet converging molecular pathways. Specific virulence factors vary among pathogens. *C. perfringens* produces the pore‐forming NetB toxin (Ou et al. 2024), *Salmonella* delivers effectors through the type III secretion system (Galán 2021), and pathogenic *E. coli* deploys the EspF protein (Singh et al. 2018). Despite this diversity, their ultimate impact converges on the disruption of TJ complexes. These pathogens trigger host inflammatory responses and activate intracellular signaling cascades, including the myosin light chain kinase (MLCK) pathway, leading to actin cytoskeleton rearrangement and the internalization or degradation of key TJ proteins, thereby increasing paracellular permeability (Rogers et al. 2023).

The protective effects of phages on the intestinal barrier have been directly validated in multiple animal models and cell culture experiments. In *Salmonella Enteritidis*‐infected chicken intestinal epithelial cells, bacteriophage treatment reduced pathogen adhesion and invasion, upregulated the expression of TJ genes (occludin, claudin‐1, ZO‐1), and suppressed pro‐inflammatory mediators (nuclear factor kappa B (NF‐κB) p65, tumor necrosis factor‐alpha (TNF‐α), interleukin‐6 [IL‐6]), collectively preserving barrier integrity (Xie et al. 2024). In a post‐weaning diarrhea model in piglets induced by enterotoxigenic *E. coli* K88, bacteriophage therapy significantly improved intestinal integrity, as evidenced by increased jejunal villus height and villus height‐to‐crypt depth ratio, reduced serum levels of intestinal permeability markers including diamine oxidase (DAO) and d‐lactate, and upregulated jejunal ZO‐1 protein expression (Chen et al. 2026).

The primary indirect mechanism by which phages protect the intestinal barrier is through targeted pathogen elimination. By significantly reducing intestinal pathogen loads, phage treatment attenuates the excessive release of pro‐inflammatory cytokines (TNF‐α, IL‐6), thereby interrupting the inflammatory cascade that drives tight junction disruption and restoring the microenvironment necessary for barrier stabilization and repair (Dong et al. 2024). Phage‐mediated pathogen reduction also restores ecological balance, allowing butyrate‐producing commensals to flourish and further reinforcing barrier integrity via short‐chain fatty acids (SCFAs)‐mediated signaling pathways (Clavijo et al. 2022).

In summary, phages protect intestinal barrier integrity primarily through targeted pathogen elimination, thereby preventing direct tight junction disruption and attenuating pathogen‐triggered inflammatory cascades. These protective effects have been validated in multiple livestock models, demonstrating preserved tight junction protein expression, improved epithelial morphology, and reduced intestinal permeability following phage treatment.

### 3.3 Modulation of Gastrointestinal Microbiota

The intestinal microbiota constitutes a highly interconnected ecological network characterized by complex competitive, mutualistic, and metabolic interdependencies among microbial populations. When pathogenic bacteria such as *E. coli*, *Salmonella*, or *C. perfringens* proliferate excessively, they not only directly damage the host but also suppress the growth of beneficial bacteria through mechanisms including secretion of antimicrobial substances and competitive exclusion, ultimately leading to intestinal dysbiosis. In this context, the targeted elimination of pathogens by phages carries significance beyond direct bactericidal activity, potentially exerting profound effects on the overall intestinal microbiota structure through cascading ecological interactions.

By selectively eliminating intestinal pathogens, phages are expected to liberate competitive ecological niches, creating favorable conditions for the recovery and proliferation of beneficial commensal bacteria and thereby facilitating the restoration of intestinal microecological balance (Rasmussen et al. 2020). The outcomes of such niche liberation are not invariably beneficial. Niches vacated by eliminated pathogens may be occupied by beneficial bacteria such as *Lactobacillus* and *Bifidobacterium* (Bao et al. 2022), but a theoretical risk of opportunistic pathogen expansion also exists, and this risk may vary with the intestinal microecological background of the host (Kanangat and Skaljic 2021). It should also be noted that most evidence for these ecological cascades in livestock and poultry comes from 16S rRNA amplicon surveys of relative abundance rather than from direct quantitative measurement.

In broiler production, the *Salmonella*‐targeting phage cocktail SalmoFree significantly reduced pathogen abundance while enriching beneficial taxa including butyrate‐producing bacteria, without disrupting the normal structure and core composition of cecal microbiota (Clavijo et al. 2022). In weaned piglets, the weaning period represents a critical phase during which the intestinal microbiota is most vulnerable to dysbiosis, often accompanied by proliferation of potentially pathogenic bacteria and reduction of beneficial bacteria. The phage vB_EcoS_ULIM2, selected against enterotoxigenic *E. coli* (ETEC), precisely targeted pathogenic bacteria while effectively preserving normal microbiota composition, indicating favorable microbiota safety in the piglet gut (Navez et al. 2023). A further study demonstrated that dietary supplementation with ETEC K88‐specific phage preparation effectively corrected intestinal microecological disturbances in diarrheic weaned piglets, significantly increased the Shannon diversity index, markedly enriched beneficial taxa including *Patescibacteria*, *Muribaculaceae*, and *Subdoligranulum*, and simultaneously reduced the relative abundance of *Proteobacteria* and *Enterobacteriaceae* (Chen et al. 2026).

Beyond monogastric animals, phages play an important role in the complex gastrointestinal microecosystem of ruminants, particularly the rumen. A study integrating rumen metagenomics and metabolomics analysis across different lactation stages in dairy cows revealed that the rumen phageome and bacteriome exhibit coordinated dynamic succession throughout lactation (Wang et al. 2025). This study confirmed that key phages can reshape rumen microbial community structure through targeted lysis of specific host bacteria, consequently modulating carbohydrate and amino acid metabolic pathways. This microecological restructuring regulated the production of volatile fatty acids (VFAs), particularly propionate, and other metabolites in the rumen, ultimately enhancing milk protein synthesis efficiency and overall lactation performance (Wang et al. 2025). Furthermore, this regulatory function may extend beyond conventional bacterial lysis to include metabolic reprogramming of host bacteria through expression of auxiliary metabolic genes (AMGs) (Yu et al. 2025). These findings highlight the phages in improving host metabolic health and production performance through maintaining gastrointestinal microecological homeostasis.

Notably, the effects of phages on intestinal microbiota do not invariably produce significant positive modulatory outcomes. Studies have confirmed that subchronic oral exposure to lytic phages targeting *Salmonella* exerted no significant effects on probiotic populations including *Lactobacillus* and *Bifidobacterium* in mouse intestines, indicating favorable microbiota safety of bacteriophages (Naveen Kumar et al. 2025). Studies have also demonstrated that intermittent bacteriophage administration regimens failed to produce sustained effective modulation of gut microbiota in healthy broilers, with minimal alteration of intestinal microbial composition (Dlamini et al. 2023). Therefore, long‐term monitoring and systematic evaluation of microbiota dynamics following phage intervention are essential for optimizing application strategies and ensuring intervention efficacy.

These divergent outcomes should be interpreted in light of the methodological constraints of the underlying evidence. Current evidence comes mostly from 16S rRNA amplicon surveys in small, single‐site controlled trials, and datasets from large‐scale commercial farms remain scarce, the SalmoFree field study being a notable exception (Clavijo et al. 2022). Across these trials, reduced target pathogen loads were the most consistent outcome. However, effects on alpha diversity were inconsistent, with some studies reporting increased Shannon indices (Chen et al. 2026) and others finding no significant impact on the overall gut microbiota following phage administration in a human gut model (Laforêt et al. 2023). Reported treatment–control separations usually rely on beta‐diversity metrics, but their magnitude and significance can shift with the distance metric and processing pipeline (Sinha et al. 2017). Retrospective power analyses of 16S data, including animal datasets, show that commonly used diversity metrics differ widely in sensitivity, so small group sizes combined with selective metric reporting can yield significant yet poorly reproducible separations (Kers and Saccenti 2022). A further caveat is intrinsic to relative abundance data. Because compositional profiles sum to a fixed total, depletion of the target pathogen mathematically inflates the relative abundance of the remaining taxa, and apparent enrichment of beneficial bacteria can therefore arise without any absolute growth of those populations (Gloor et al. 2017; Vandeputte et al. 2017). Niche liberation is therefore plausible, but it remains an inference until tested with absolute quantification in adequately powered studies. Future studies would be strengthened by absolute quantification of key taxa, exact sequence variant processing, compositional data analysis, and standardized reporting in replicated commercial farm trials (Knight et al. 2018; Mirzayi et al. 2021).

Taken together, the modulatory effects of phages on the intestinal microbiota of livestock and poultry fundamentally derive from the ecological restructuring triggered by precise pathogen elimination. The most robust conclusion supported by current 16S evidence is that phage intervention preserves overall community diversity and structure, in clear contrast to the nonspecific disruption caused by broad‐spectrum antibiotics. Whether phages also drive consistent, directional enrichment of beneficial taxa through niche liberation remains an open question, because the supporting signals are heterogeneous across studies and originate mainly from small controlled trials analyzed with non‐standardized pipelines. The direction and magnitude of ecological cascade effects are further influenced by the host intestinal background, the administration regimen, and phage characteristics. Confirmation at commercial farm scale, using absolute quantification and harmonized bioinformatic standards, will be essential before this mechanism can be considered established for specific application scenarios.

### 3.4 Modulating Host Immunity

The intestinal tract serves not only as the primary organ for nutrient digestion and absorption, but also as one of the most immunologically significant tissues in the body. The gut‐associated lymphoid tissue (GALT) represents one of the largest immune organs, harboring a substantial concentration of immune cells that play central roles in host defense and immune regulation (Bemark et al. 2024). The intestinal microbiota and host immune system engage in a dynamic, bidirectional relationship through what is conceptualized as the gut‐immune axis (Ciaușu‐Sliwa et al. 2026). Under homeostatic conditions, commensal microbiota and the host immune system maintain a harmonious symbiotic relationship, collectively establishing a sophisticated balance between immune tolerance and defense (Arenas‐Gómez et al. 2023). However, excessive proliferation of pathogenic bacteria disrupts this equilibrium, triggering persistent intestinal inflammation that subsequently disturbs systemic immune homeostasis (Wozniak et al. 2022). Through their unique biological characteristics, phages play important roles in modulating host immune responses through mechanisms that encompass both indirect immunomodulation via regulation of pathogen abundance and potential direct interactions with the immune system (Sinha and Maurice 2019).

The indirect immunomodulatory effects of phages are primarily manifested through effective control of local intestinal inflammatory responses. Upon infection with intestinal pathogens such as *Salmonella*, *Clostridium perfringens*, or pathogenic *E. coli*, pathogen‐associated molecular patterns (PAMPs), including LPS and flagellin, released by these bacteria are recognized by pattern recognition receptors within the GALT, particularly Toll‐like receptors (TLRs), which subsequently activate downstream signaling cascades including NF‐κB, thereby inducing robust pro‐inflammatory responses (Kawai et al. 2024). While this immune activation serves a protective function during the initial stages of infection, sustained excessive inflammation leads to tissue damage and immune dysfunction (Yin et al. 2023). By reducing intestinal pathogen burdens and the subsequent release of PAMPs, phages attenuate excessive TLR/NF‐κB signaling activation, thereby producing net anti‐inflammatory benefits while preserving intestinal barrier integrity (Dong et al. 2024). Furthermore, a comparative experimental study reported that enrofloxacin treatment was associated with cytokine imbalance, immune‐cell dysregulation, and stress‐axis hyperactivation, whereas bacteriophage therapy attenuated aberrant increases in pro‐inflammatory cytokines, including interleukin‐1β (IL‐1β), interleukin‐6 (IL‐6), interferon‐γ (IFN‐γ), interleukin‐8 (IL‐8), and interleukin‐12 (IL‐12), and increased the anti‐inflammatory cytokines interleukin‐10 (IL‐10) and interleukin‐4 (IL‐4) (Grabowski et al. 2022). Bacteriophage therapy also did not adversely affect lymphocyte subset counts or activity, suggesting a more favorable immunological safety profile than enrofloxacin under the tested conditions. However, because the immunomodulatory effects of antibiotics vary substantially across drug classes, dosages, host species, infection models, and treatment regimens, these findings should not be extrapolated to all veterinary antibiotics.

The immunomodulatory effects described above are primarily attributable to phage‐mediated reduction of pathogen burden. Notably, emerging evidence suggests that phages may also exert immunomodulatory functions through direct interactions with host immune cells, independent of their bactericidal activity, opening new dimensions in phage immunology research. Although phages cannot undergo productive replication in eukaryotic cells, substantial evidence indicates that phage particles can be internalized by intestinal epithelial cells and antigen‐presenting cells, including macrophages and dendritic cells within the lamina propria (Gembara and Dąbrowska 2024). For instance, certain phages have been found to induce interferon or cytokine production through interactions with Toll‐like receptors, thereby modulating host immune responses (Carroll‐Portillo et al. 2025). A study has suggested that in chickens infected with *Salmonella enterica* and treated with a phage cocktail, phages were initially recognized by host cells as virus‐like agents. However, downstream antiviral signaling was attenuated because the Cyclic GMP‐AMP synthase–stimulator of interferon genes (cGAS‐STING)‐mediated response was blocked at the level of interferon regulatory factor 3 (IRF3) phosphorylation. This blockade was attributed to the failure of RNA polymerase III to recognize phage DNA and generate dsRNA intermediates required for IRF3 activation, thereby limiting excessive inflammatory activation (Podlacha et al. 2024). Additionally, phage particles themselves may function as adjuvant‐like immunostimulatory structures, potentially enhancing host recognition and clearance of pathogenic bacteria. Ex vivo assays with primary bovine blood cells showed that immune recognition of phages is phage dependent, with some phages eliciting strong immune responses and others evading recognition (Démoulins et al. 2025). This direct immunomodulatory activity may have dual consequences. Although it may enhance antibacterial defense through adjuvant‐like mechanisms (Al Allaf et al. 2026), it may also elicit anti‐phage immune responses, such as neutralizing antibodies or accelerated phage clearance, that could reduce therapeutic efficacy (Berkson et al. 2024). The net balance between beneficial immunostimulation and potentially detrimental anti‐phage immunity should therefore be evaluated through systematic in vivo investigations. Overall, these findings suggest that phage‐mediated immune effects extend beyond pathogen reduction and microbiota‐mediated mechanisms to include direct, pathway‐specific interactions with host cells, which should be considered when evaluating the immunological basis and therapeutic performance of phage therapy (Souza et al. 2023).

In summary, phages regulate host immune responses through multiple pathways. On the one hand, they precisely eliminate pathogenic bacteria, reduce sustained PAMP stimulation, and effectively control excessively activated inflammatory responses. On the other hand, they may directly affect the functional status of immune cells via potential interactions with antigen‐presenting cells. This immunomodulatory mode, which combines burden alleviation and immune modulation, enables phages to control pathogenic infections while maintaining intestinal immune homeostasis.

## 4 Key Experimental Evidence for Phages in Improving Animal Intestinal Health

While the preceding mechanisms outline potential pathways for phage‐mediated intestinal protection, their practical significance requires careful experimental scrutiny. Evidence from controlled trials in broilers, piglets, and ruminants indicates that phage intervention may improve intestinal integrity and microbial balance, with associated gains in growth performance and disease resistance. Nevertheless, outcomes vary with administration route, dosage, timing, and formulation, factors that have typically been studied independently rather than in combination. Furthermore, most findings originate from laboratory or specific challenge models, and broader field consistency remains to be systematically established. Figure 2 schematically integrates these application variables with proposed intestinal mechanisms and their reported outcomes, offering a conceptual framework that reflects current experimental evidence.

**Figure 2:**
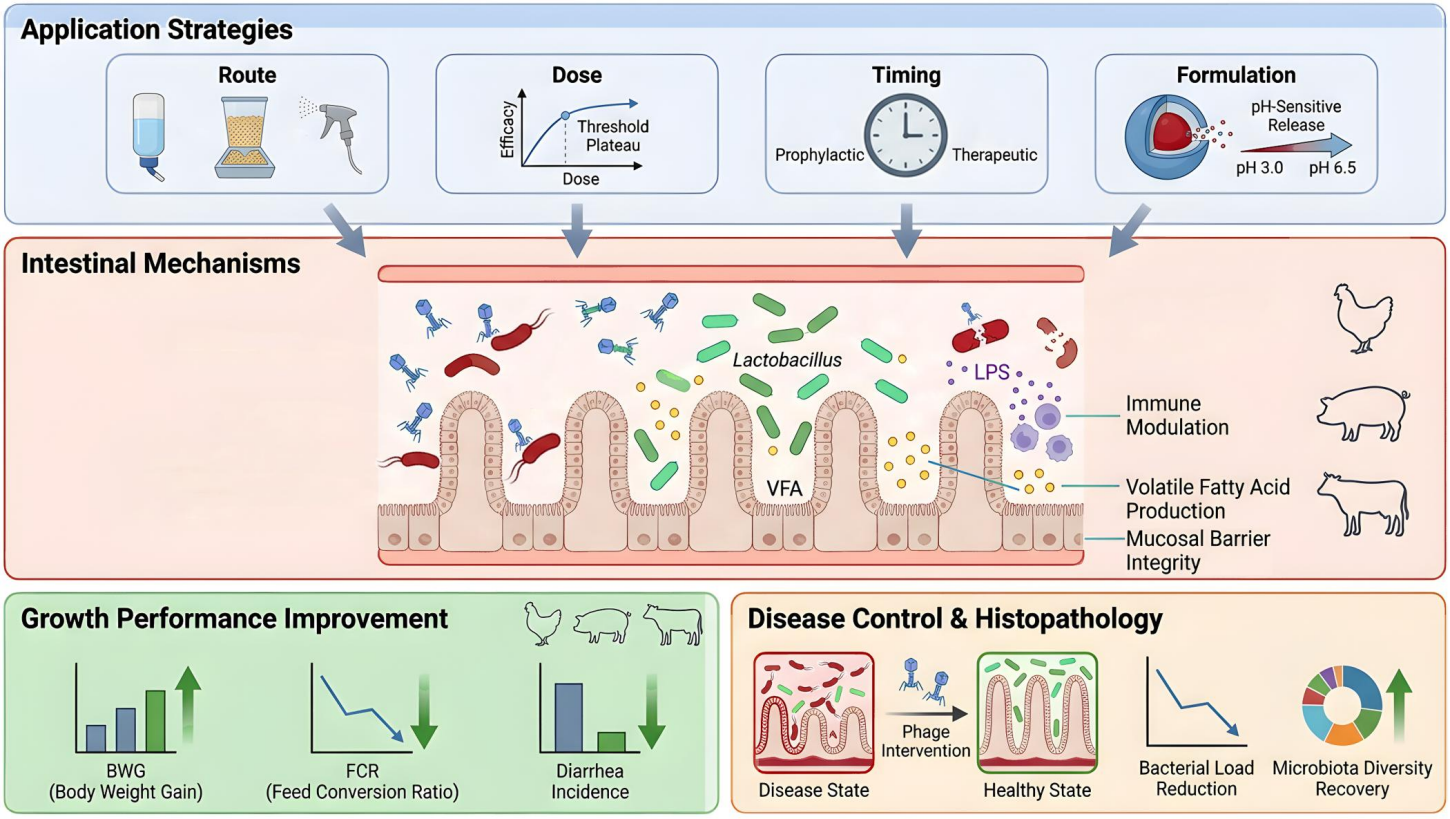
Integrated framework of bacteriophage application strategies and intestinal health outcomes in livestock and poultry. Bacteriophage‐mediated intestinal protection spans interconnected application, mechanistic, and phenotypic dimensions. At the application level, delivery route, dose‐response thresholds, intervention timing, and formulation technologies influence phage delivery efficiency. At the intestinal level, phages may reduce pathogen loads, support beneficial commensals such as *Lactobacillus*, decrease lipopolysaccharide (LPS) release, enhance volatile fatty acid (VFA) production, modulate local immune responses, and contribute to mucosal barrier integrity. At the outcome level, reported responses include improved body weight gain (BWG), reduced feed conversion ratio (FCR), decreased diarrhea incidence, restoration of intestinal morphology, lowered bacterial burden, and recovery of microbiota diversity. Together, these dimensions outline the proposed translation of strategic phage application into intestinal health benefits.

### 4.1 Growth Performance Improvement Driven by Intestinal Health

Intestinal health is closely associated with the growth performance of livestock and poultry. A healthy intestine is characterized by efficient digestion and absorption of nutrients, a stable microbial community structure, and normal immune function, which collectively determine feed conversion efficiency and growth rate (Walter et al. 2025). By maintaining intestinal health, phages indirectly improve growth performance.

In Ross 308 broilers, phage supplementation in a 35‐day high‐ and low‐protein feeding trial increased final body weight (BW) and cumulative body weight gain (BWG), improved nitrogen digestibility, and enhanced ileal microbial diversity under the low‐protein diet, suggesting that phages can complement protein‐reduction strategies for more sustainable poultry production (Sampath et al. 2026). Dietary supplementation with a bacteriophage cocktail similarly improved BWG, feed efficiency, cecal microbial composition, and production traits in broiler chickens (Upadhaya et al. 2021). In weaned piglets, dietary supplementation with phages significantly increased final body weight, average daily gain, and average daily feed intake, while markedly reducing feed‐to‐gain ratio and diarrhea incidence (Zeng et al. 2021). These improvements were closely related to optimized intestinal morphology, regulated serum immunoglobulins and cytokines, enhanced ileal mucosal barrier function, and improved cecal microbial diversity, which confirmed the mechanism by which phages promote the growth of weaned piglets through regulating intestinal health (Zeng et al. 2021). In ruminant animals, emerging strategies utilize viral agents, including phages targeting hydrogen‐producing bacteria and archaeal viruses targeting methanogenic archaea, to specifically mitigate methanogenesis (Khairunisa et al. 2023; Magossi et al. 2025). By selectively modulating these key microbial populations, phage interventions can redirect metabolic hydrogen flux away from methane formation towards the production of volatile fatty acids, thereby reducing energy loss and greenhouse gas emissions (Yu et al. 2025). This optimization of rumen fermentation efficiency has the potential to enhance feed conversion rates and direct more energy and nitrogen sources towards the host animal, fundamentally supporting improved productivity (Xue et al. 2024).

In summary, by maintaining intestinal microbial balance, enhancing mucosal barrier integrity, and optimizing nutrient metabolism, phages consistently improve growth performance across poultry, swine, and ruminants. These improvements manifest as increased body weight gain, enhanced feed conversion efficiency, and reduced pathological symptoms, demonstrating that phage‐mediated intestinal health translates directly into measurable productivity benefits.

### 4.2 Prevention and Control of Intestinal Diseases and Improvement of Histopathology

Intestinal health is directly reflected by the ability to resist diseases. The preventive and therapeutic effects of phages on intestinal diseases in livestock and poultry can be quantitatively evaluated via histopathology. Such evidence directly validates the practical efficacy of phages in maintaining intestinal health.

In a *Clostridium perfringens*‐induced necrotic enteritis broiler model, oral bacteriophage administration significantly reduced intestinal lesion scores in the duodenum and jejunum to levels comparable to uninfected healthy controls, effectively protecting broiler intestinal integrity (Keerqin et al. 2022). In weaned piglets infected with *Salmonella Typhimurium*, phage STP‐1 treatment reduced bacterial colonization in feces and intestinal tissues, increased jejunal villus height and goblet cell density, and alleviated intestinal structural damage. Consequently, it improved average daily gain, thereby mitigating the growth inhibition and intestinal injury caused by *Salmonella* (Won et al. 2021). In a weaned piglet model of Shiga toxin‐producing *E. coli* (STEC)‐induced edema disease, prophylactic oral administration of phage P‐GXEC‐L2P5 prevented clinical signs and intestinal pathology, reduced fecal STEC shedding and systemic Shiga toxin levels, and preserved jejunal microbiota diversity, demonstrating comprehensive intestinal pathogen control and disease prevention (Ma et al. 2025).

In summary, histopathological evidence from multiple disease models confirms that phages effectively prevent and treat intestinal infections in livestock and poultry. By reducing pathogen colonization, restoring intestinal morphology, and preserving microbiota diversity, phages mitigate disease‐associated intestinal damage and growth inhibition. These findings collectively validate the practical efficacy of phage‐based interventions in maintaining intestinal health and protecting animal productivity.

### 4.3 Effects of Application Strategies on Intestinal Health

The protective efficacy of phages in maintaining intestinal health in livestock and poultry depends not only on the biological characteristics of the phages themselves but also closely on specific application strategies. Different administration routes, dosages, timings, and formulation forms affect the survival rate, the targeting efficiency of phages in the gastrointestinal tract, and their ultimate beneficial effects on intestinal health. Sorting out these influencing factors is of great practical significance for optimizing the clinical application of phage products.

Administration route is one of the key factors affecting phage efficacy. The main administration methods in livestock and poultry production include oral routes such as drinking water, feed mixing, and gavage, as well as environmental spraying (Mehmood Khan et al. 2023). In addition, phage inoculation into the allantoic fluid of chicken embryo represents a promising early prophylactic strategy, effectively protecting hatched chicks against *avian pathogenic Escherichia coli* (APEC) O2 challenge through reduced intestinal bacterial loads and prevention of colibacillosis lesions (Nicolas et al. 2023).

The dose–effect relationship is a critical parameter to be clarified in phage application research. Animal experiments have shown that the antibacterial efficacy of phages is generally dose‐dependent, but not in a simple linear manner. Once the phage dosage reaches a certain threshold, further increasing the dose does not proportionally enhance bacterial clearance, which may be related to limited diffusion of phages in the intestine and the accessibility of target bacteria (Zeng et al. 2021). Notably, low‐dose or low‐activity phages may even induce bacterial resistance under certain conditions (Torres‐Barceló et al. 2022). Therefore, determining the optimal therapeutic dose is essential for both clinical efficacy and resistance management.

The timing of intervention significantly affects the protective effect of phages on intestinal health. Distinct differences exist between prophylactic and therapeutic administration. In an SPF broilers chicken model of *Salmonella Pullorum* infection, prophylactic administration of phage vB_SalS_JNS02 was significantly superior to delayed therapeutic dosing, more effectively reducing bacterial loads in blood and tissues, accelerating pathogen clearance from the brain and blood, and restoring intestinal morphology while alleviating tissue damage (Li et al. 2025). This advantage is attributed to lower bacterial loads, enhanced target cell accessibility, and the absence of biofilm formation or host cell invasion during the early stage of infection; conversely, therapeutic administration, though partially effective, is constrained against pathogens that have already established intracellular residence. However, the durability of prophylactic protection is not guaranteed. In a broiler model, administration of a six‐phage cocktail via drinking water for 6 days post‐hatching significantly reduced cecal *Salmonella* colonization within 4 days after challenge on day 7; yet bacterial levels rebounded 8 days after phage withdrawal (Agapé et al. 2024). Thus, while phage prophylaxis can initially control infection, administration regimens require optimization to sustain protection throughout the production cycle.

The influence of formulation technology cannot be ignored. In a porcine intestinal epithelial cell model infected with ETEC EC43‐Ph, calcium carbonate/alginate‐microencapsulated phage FJ1 survived simulated gastric fluid (pH 3.0) and was activated at intestinal pH (6.5), reducing ETEC by approximately 99.9% within 6 h; notably, although bacteriophage‐insensitive mutants emerged, they incurred fitness costs including increased complement susceptibility and reduced colonization capacity (Ferreira et al. 2023). In a weaned piglet model of *E. coli*‐induced diarrhea, oral administration of microencapsulated phage A221 significantly increased daily weight gain, reduced Enterobacteriaceae abundance in the duodenum to 0.64%, decreased bacterial loads in mesenteric lymph nodes, cecum, and spleen, and ameliorated histopathological damage in the cecum and duodenum, achieving therapeutic efficacy comparable to florfenicol (Mao et al. 2023). Dietary supplementation with Eudragit L100 spray‐dried microencapsulated phage FGS011 provided superior protection against gizzard harsh conditions and enabled efficient intestinal and cecal delivery throughout the 42‐day broiler production cycle compared with free phage administered via drinking water (Lorenzo‐Rebenaque et al. 2022).

In summary, phage efficacy in livestock and poultry intestinal health depends on the integrated optimization of route, dosage, timing, and formulation. However, current studies largely examine these factors in isolation, and the parameter combinations that perform best under real‐field conditions have yet to be defined across diverse production systems. This knowledge gap underscores the need for multi‐parameter optimization frameworks in future phage application protocols.

## 5 Current Challenges and Future Perspectives

### 5.1 Efficacy Limitations and Failure Analysis

Controlled challenge studies in livestock and poultry predominantly report positive outcomes. However, a recent systematic review has revealed substantial heterogeneity in efficacy across studies (Rahman et al. 2026). This subsection critically appraises representative failed, limited, and non‐durable in vivo phage trials in livestock and poultry.

Rapid clearance and insufficient exposure at the infection site constitute the first major cause of failure. Orally administered free phages must survive gastric acidity, bile salts, and proteolytic enzymes before reaching the distal intestine, while phages entering the circulation are rapidly sequestered by the reticuloendothelial system (Merril et al. 1996; Dąbrowska and Abedon 2019). Critically, the administered dose is a poor proxy for site exposure. In feedlot steers, groups shedding higher mean fecal phage titers did not differ from controls in the number of *E. coli* O157:H7‐positive samples, despite receiving up to 4.8 × 10^11^ plaque‐forming units (PFU) in repeated oral and/or rectal doses (Rozema et al. 2009). Moreover, the administration route determines whether effective titers reach the target tissue at all. In a respiratory colibacillosis model, spray application of phage failed to protect 1‐day‐old broilers against intratracheal *E. coli* challenge, whereas intratracheal administration was completely protective (Huff et al. 2013). These cases indicate that failure often reflects insufficient active phage at the site of infection rather than inadequate administered doses.

Anti‐phage immune responses represent a second cause of failure. Phage particles are actively cleared by host immunity, including phagocytes, complement, and antibodies (Bosco et al. 2026). Direct evidence in poultry demonstrates that such responses can compromise efficacy. Pre‐immunization of broilers with phage TM1 reduced the efficacy of subsequent TM1 treatment against colibacillosis, whereas the less immunogenic phage TM3 was unaffected, indicating that serum anti‐phage activity is phage‐type dependent (Naghizadeh et al. 2019). In mice, gut secretory IgA induced by prolonged oral administration antagonized the intestinal transit of active phage and was recalled more rapidly upon re‐exposure (Majewska et al. 2015), a timeframe relevant to repeated dosing over livestock production cycles.

Inappropriate dosing relative to pharmacokinetic thresholds is a third cause of failure. As self‐amplifying agents, phages obey threshold pharmacology that below critical phage‐to‐bacterium ratios at the infection site, in situ amplification fails and clearance can be achieved only by passive inundation (Danis‐Wlodarczyk et al. 2021). Clearance of intestinal *E. coli* O157:H7 in mice required phage/bacterium ratios of ≥ 10^2^, and a cattle protocol designed to achieve such ratios at the recto‐anal junction reduced, but did not eliminate, bacterial carriage (Sheng et al. 2006). Higher doses are also not necessarily superior. In broilers, oral administration at multiplicity of infection (MOI) of 1 reduced *Salmonella* shedding more effectively than MOI 2, whereas in ovo administration at MOI 2 produced the most sustained suppression, indicating route‐dependent and non‐linear dose–response relationships (Kawthekar M et al. 2026).

Spatial exclusion, ecological mismatch, and field‐level confounders constitute the fourth category. Enterohemorrhagic *E. coli* O157:H7 preferentially colonizes the recto‐anal junction (RAJ) of ruminants (Wells et al. 2020). Phage KH1 inhibited *E. coli* O157:H7 in vitro, but oral administration alone failed to reduce intestinal carriage in sheep, whereas direct application to the RAJ mucosa achieved significant yet incomplete reduction in cattle at ratios ≥ 10^2^ (Sheng et al. 2006). Ecological mismatch arises when phages selected against limited strain panels encounter diverse field populations. Commercial broiler flocks harbor genetically distinct *Campylobacter jejuni* populations that differ between farms and turn over during the production cycle (Truccollo et al. 2021), whereas most phage recognition in *Campylobacter* is strain‐specific, with the primary isolation strain determining both phage type and the receptors recognized (Sørensen et al. 2015). Field outcomes bear the expected signature of this mismatch, being variable between flocks and short‐lived, as a *Campylobacter* phage cocktail effective under controlled conditions significantly reduced cecal loads in only one of three commercial flocks (Kittler et al. 2013). A phage mixture applied to naturally contaminated broilers reduced fecal counts by only up to 1.1 log_10_ CFU/mL 1 day after dosing, with no evidence of synergy when combined with other interventions (Bogun et al. 2024), and even under controlled conditions, cecal reductions peaked 2 days post‐treatment before attenuating (Richards et al. 2019). Nor does cocktail formulation fully resolve the mismatch, because in vitro, combinations of O157‐specific phages proved neutral, facilitative, or antagonistic depending on phage genera, bacterial strain, MOI, and temperature (Niu et al. 2021), indicating that cocktail performance in vitro cannot be assumed to predict efficacy in the field. A further confounder is the endogenous phage background of food animals. Resident phages can interfere with the binding of therapeutic phages to their target bacteria, and together with the resident bacterial community they constrain the adsorption, replication, and lytic activity of administered phages, so that trial outcomes partly reflect background phage activity rather than the administered preparation (Rahman et al. 2026). Bovine feces harbor diverse bacteriophages with lytic activity against non‐O157 Shiga toxin‐producing *E. coli*, some of which can also lyse common phage types of STEC O157:H7, supporting the existence of natural phage reservoirs in livestock‐associated environments (Wang et al. 2015). In *E. coli* O157:H7 phage therapy trials in sheep and cattle, phages distinct from the administered therapeutic phages emerged after bacterial inoculation despite not being detected before the trials (Kropinski et al. 2012). Together, these findings suggest that naturally occurring or unintentionally introduced phages may obscure treatment effects, and complicate efficacy assessment under field conditions.

In synthesis, the causes of failure are diverse and often intertwined, but several themes recur across otherwise unrelated studies, including insufficient delivery of active phages to the site of infection, host immune clearance, inappropriate dosing relative to pharmacodynamic thresholds, and spatial exclusion coupled with ecological mismatch. Future trials would therefore benefit from pre‐trial surveillance of resident target strains, from reporting phage exposure at the infection site rather than administered doses alone, from monitoring anti‐phage immunity during repeated administration, and from testing all groups, including untreated controls, for endemic phages.

### 5.2 Phage Resistance and Countermeasures

Bacteriophage resistance represents an inevitable biological challenge in phage therapy. Through long‐term co‐evolution, bacteria have evolved multiple defense strategies, either by mutating or modifying surface receptors (e.g., LPS, outer membrane proteins, and pili) to block phage adsorption (Hasan and Ahn 2022), or by activating intracellular defense systems (e.g., restriction‐modification, CRISPR‐Cas, and abortive infection systems) to degrade foreign nucleic acids and prevent progeny phage release (Arias et al. 2022). Moreover, in vivo studies indicate that intestinal resistance dynamics can be further complicated by alternative genetic mechanisms, such as plasmid‐mediated phage interference, which may substantially alter phage susceptibility under practical conditions (López‐Pérez et al. 2023). These findings collectively suggest that the intestinal environment poses unique challenges to sustained phage efficacy.

Notably, the development of phage resistance in bacteria is often associated with fitness costs. For example, LPS synthesis defects in phage‐resistant *E. coli* reduce adherence to and colonization of host epithelial cells (Zulk et al. 2022); *rfbD* mutations in phage‐resistant *Salmonella Enteritidis* decrease growth rates and increase susceptibility to environmental stress and antibiotics (Zeng et al. 2024); and cell membrane protein alterations in phage‐resistant *Stenotrophomonas maltophilia* are accompanied by reduced virulence, weakened biofilm formation, and enhanced aminoglycoside sensitivity (Han et al. 2024). These findings suggest that even if resistant strains emerge during therapy, their pathogenicity and competitive fitness may be concurrently diminished, thereby mitigating clinical risks to some extent. However, fitness costs are not always significant and vary substantially depending on the specific gene and receptor involved (Wu et al. 2026). Some resistant mutants even exhibit limited fitness costs with minimally affected growth rates (Cai et al. 2022). Moreover, bacteria may gradually alleviate incurred costs through long‐term coevolution, enabling resistant phenotypes to stabilize over time (Xu et al. 2023). The universality of these specific mechanisms therefore remains to be systematically validated in additional livestock and poultry pathogen–phage systems.

In practical applications, bacteria frequently develop resistance to single phages; therefore, cocktail strategies combining multiple phages targeting different receptors are adopted to constrain the emergence of resistant strains, as simultaneous resistance to multiple targets is highly unlikely. When bacteria evade one phage by altering a specific receptor, they remain susceptible to other components targeting different receptors and are effectively lysed (Yang et al. 2020). Against multidrug‐resistant *E. coli*, phage cocktails significantly enhance antibacterial activity and suppress the emergence of resistant strains, outperforming single‐phage treatment (Zulkarneev et al. 2025). In *Salmonella*, phage cocktails targeting multiple receptors including O‐antigen, BtuB, and OmpC exhibit significantly lower frequencies of bacteriophage‐insensitive mutants than single phages, indicating that single‐receptor mutations cannot simultaneously evade lysis by all components (Martinez‐Soto et al. 2024). Furthermore, rationally designed cocktails can direct the evolution of resistant mutants toward more severe fitness costs through multi‐receptor selective pressure, characterized by reduced virulence and increased antibiotic sensitivity (Gao et al. 2022). Beyond cocktails, phage‐antibiotic synergy (PAS) represents an important complementary strategy for delaying resistance (Anastassopoulou et al. 2024). However, such combinations may produce either synergistic enhancement or antagonistic effects (Supina and Dennis 2025). Additionally, phage‐derived proteins encoded by lysis cassette genes, such as endolysins, holins, and spanins, can be applied directly as therapeutic candidates, representing an alternative approach distinct from live phage therapy (Aslam et al. 2021).

Current evidence demonstrates that cocktail strategies effectively delay resistance development through multi‐receptor targeting; however, their efficacy is highly dependent on rational pairing of phage receptor specificities, and the dynamic nature of fitness costs introduces uncertainty in long‐term predictions. Additionally, the synergistic or antagonistic effects of PAS and the field application potential of enzymatic agents remain inadequately validated in livestock systems. Future research should establish standardized platforms for resistance monitoring and phage screening, and optimize combination strategies for specific farming contexts, to facilitate the translation of laboratory findings into stable field applications.

### 5.3 Phage Delivery Efficiency and Formulation Technology

Oral delivery exposes phages to a series of gastrointestinal barriers before they reach the distal intestine. Gastric acid, proteolytic enzymes, bile salts, and the mucus layer each reduce the active fraction of the dose (Zelasko et al. 2017; Dlamini et al. 2023). What these barriers look like, and how severe they are, depends on the species. A phage delivery system that performs well in one animal can fail in another, and formulations need to be judged against the gastrointestinal tract of the intended host (de Gennaro et al. 2026).

In poultry, feed passes from the crop to the acid–pepsin proventriculus, then to the muscular gizzard for mechanical grinding before intestinal entry (Rodrigues and Choct 2018). This combination of acid and mechanical shear is hard on unprotected phages. Under in vitro conditions mimicking the proventriculus and gizzard, free *Salmonella* phages were inactivated, whereas phages microencapsulated in pH‐responsive Eudragit polymers survived (Lorenzo‐Rebenaque et al. 2021). This protection was confirmed in vivo in broilers (Lorenzo‐Rebenaque et al. 2022). However, protection alone is insufficient because the time available for release is short. Solid digesta remains in the broiler gizzard for only about 12 to 34 min depending on the diet (Garçon et al. 2023), and a matrix that is too robust may pass through the target segments before releasing its payload.

The pig stomach presents a different problem. It is a single glandular chamber in which acid and pepsin dominate, and mechanical stress amounts to little more than mixing contractions. The challenge there is prolonged chemical exposure. In vitro simulations of this sustained acid stress show that appropriately engineered capsules can meet the challenge. CaCO_3_‐supplemented alginate–carrageenan microcapsules limited phage loss to < 0.5 log_10_ PFU/mL after 1 h at pH 2, compared with complete inactivation of free phage within 10 min below pH 3, and released their cargo efficiently once the pH was raised to neutral (Dlamini et al. 2023). Nor do the demands end at the pylorus. Once in the small intestine, the matrix must break down in a controlled manner to ensure timely release in the intestinal lumen. A pH‐responsive core–shell design showed comparable tolerance down to pH 1, releasing its cargo within 10–30 min following a pH shift representative of the lower gastrointestinal tract in vitro (Richards and Malik 2021).

Ruminants raise the difficulty further. An orally delivered formulation first enters the reticulorumen, a near‐neutral anaerobic chamber where material is retained for long periods within a dense microbial population capable of degrading polymer matrices. Only afterwards does it reach the acidic abomasum. Ruminant formulations therefore need rumen bypass on top of acid resistance, and here the evidence base is thin, with phage‐specific encapsulation studies largely missing (de Gennaro et al. 2026).

Several carrier systems have been developed to improve intestinal phage delivery. Microcapsules are the most extensively studied. A protective shell physically separates the phage from gastric acid and digestive enzymes, and the shell can be functionalized with pH‐responsive materials such as alginate (Gomez‐Garcia et al. 2021) or chitosan‐based composite matrices (Karthika et al. 2025) for enhanced acid tolerance and intestinal release. The avian evidence matches the demands outlined above. Spray‐dried microcapsules of the enteric polymer Eudragit L100 carried phage FGS011 through the proventriculus–gizzard environment and achieved efficient delivery to the intestine and ceca throughout the 42‐day broiler production cycle, performing better than free phage given in drinking water (Lorenzo‐Rebenaque et al. 2022). Alginate‐based microcapsules showed the same pattern. Microencapsulated *Salmonella* phages proved far more stable in simulated gastric fluid than free phages, were completely released within 4 h in simulated intestinal fluid, and gave better therapeutic outcomes than free phages in chick colonization experiments (Zhang et al. 2023). Liposomes take a different approach. Their phospholipid bilayer resembles the cell membrane, which improves mucosal adhesion, prolongs retention, and offers some protection from clearance by phagocytes (Singla et al. 2016; Otero et al. 2019). In broilers, cationic liposome‐encapsulated *Salmonella* phages lost less titer in simulated gastric fluid than free phages and maintained protective efficacy for up to 1 week after dosing stopped (Colom et al. 2015). Work in swine has concentrated on pH‐triggered release. Calcium carbonate/alginate‐microencapsulated phage FJ1 survived simulated gastric fluid at pH 3.0 and released its payload at intestinal pH 6.5, markedly reducing ETEC in a porcine intestinal epithelial cell model (Ferreira et al. 2023). Given orally to weaned piglets, microencapsulated phage A221 lowered intestinal pathogen loads and ameliorated histopathological damage (Mao et al. 2023). Beyond capsule chemistry, processing technologies add further control. Freeze‐drying (Zhang et al. 2021) and spray‐drying (Malik 2021) convert phage preparations into dried particles with improved storage stability, and pH‐responsive microencapsulation by membrane emulsification, followed by direct compression into enteric‐coated tablets, extends this control into solid oral dosage forms with site‐targeted release (Vinner et al. 2019).

These differences translate into distinct design requirements. Poultry formulations must pair mechanical robustness with fast release. Swine formulations call for lasting acid and protease protection followed by controlled release in the intestine. For ruminants, there is the added requirement of surviving ruminal fermentation.

Microcapsules, liposomes, and dried particles each improve oral phage delivery through distinct protective mechanisms and release profiles. Most of the evidence, however, comes from laboratory media or single‐species models, and long‐term stability and cost‐effectiveness across species, age groups, and field settings have yet to be evaluated systematically. Two gaps stand out. Conventional simulated gastric fluid assays do not reproduce the mechanical grinding of the avian gizzard, so current validation systems may overestimate what a capsule can survive in poultry. And for ruminants, phage‐specific encapsulation evidence is largely absent even though ruminal fermentation poses distinct challenges. Comparative testing of carrier systems in livestock models, together with screening frameworks that weigh delivery efficiency against production cost and on‐farm feasibility, would move phage formulations from laboratory development toward field application.

### 5.4 Combined Application of Phages With Non‐Antibiotic Interventions

The combined application of phages with other non‐antibiotic strategies, as a multi‐target and multi‐mechanism integrated intervention approach, is emerging as an effective means to enhance intestinal health management.

The combined use of bacteriophages and probiotics has shown promise for improving broiler performance and modulating gut microbiota. In a feed‐additive trial, supplementation with a phage cocktail plus probiotics improved BW, BWG, and feed conversion ratio (FCR) relative to the basal‐diet control, and phage probiotic groups showed greater enrichment of putative SCFA ‐producing bacteria than non‐combination groups (Shaufi et al. 2023). In *C. perfringens*‐challenged broilers, combined administration of phage SLAM_phiCP2S1 and probiotic *Pediococcus pentosaceus* SLAM_PEP01 was more effective than either monotherapy in preserving growth performance and intestinal health, reducing cecal *C. perfringens* burden, and promoting beneficial microbial taxa while reducing pathogen‐associated genera (Choi et al. 2026). In a commercial farm experiencing recurrent *Salmonella* outbreaks, a pre‐post intervention combining probiotics and phages was associated with a 90% reduction in environmental and cecal *Salmonella* detection, a 72% reduction in mortality, a 6.4% increase in BW, increased cecal microbial diversity, and earlier appearance of *Ruminococcus*, *Clostridium*, and *Faecalibacterium* (Soffritti et al. 2025). However, because the field study used a pre‐post design in an outbreak‐specific setting, randomized controlled commercial trials are still needed to confirm whether these effects are reproducible and attributable to phage probiotic complementarity.

These encouraging results should not be taken to mean that phage–probiotic combinations are automatically compatible. Host specificity lowers the risk of off‐target activity, yet beneficial bacteria may themselves be phage hosts. Dairy starter cultures have been destroyed by phages for decades despite rigorous strain selection (Romero et al. 2020), and probiotic *Bacillus* strains express surface structures such as wall teichoic acids that may act as phage receptors (Xing et al. 2025). Compatibility therefore has to be tested directly. After the usual genomic safety checks, receptor‐binding protein annotation can help prioritize candidates, but host range is also shaped by intracellular defense systems and still has to be measured experimentally (Leprince et al. 2026). Testing should cover two panels, one built from recent field isolates of the target pathogen and the other from the exact commercial probiotic strains intended for co‐administration, with representative commensals added where feasible. Activity on these panels should be scored by combining plaque or spot assays with adsorption, efficiency‐of‐plating, and liquid co‐growth measurements (Hyman 2019). Any phage showing reproducible activity against a probiotic strain should be excluded or replaced by one that recognizes a different receptor. Compatible combinations should then be tested in the target animal species, with pathogen reduction and strain‐specific persistence of the supplemented probiotic tracked as joint endpoints, before replicated evaluation on commercial farms (Shanduka et al. 2026).

The combined application of phages and plant extracts exhibits concentration‐dependent and extract‐specific outcomes. In a broiler necrotic enteritis model, dietary supplementation with 5% black cumin seed extract combined with drinking water administration of anti‐*C. perfringens* phages reduced mortality to 0%, significantly outperforming single treatments without adversely affecting growth performance (Manjunaha et al. 2026). *In vitro* investigations reveal that the interaction is highly variable. Cinnamon or clove essential oils at half of the minimum inhibitory concentration may enhance phage lytic activity (Elafify et al. 2025), whereas methanol extracts of *Echinacea purpurea* and *Ruta graveolens* at high concentrations suppress phage activity and promote bacterial growth (Stachurska et al. 2023). These findings indicate that phage‐plant extract combinations are highly context‐dependent and require case‐specific evaluation rather than generalized application.

Overall, phage–probiotic and phage–plant extract combinations remain promising multi‐target strategies for intestinal health management in livestock and poultry, but their effects should not be assumed to be uniformly additive. Phage–probiotic products require strain‐level compatibility testing among the phage cocktail, the supplemented probiotic, and the target pathogen, whereas phage–plant extract combinations require evaluation of extract‐ and dose‐dependent effects on phage stability, bacterial susceptibility, and gut microbial responses. Future studies should move beyond efficacy‐only testing and incorporate pre‐specified compatibility screens, mechanistic interaction assays, and replicated farm validation that tracks pathogen reduction, persistence of the companion additive, and production outcomes.

### 5.5 Commercial Scalability, Downstream Processing, and Cost‐Effectiveness

Laboratory efficacy does not guarantee commercial feasibility. Livestock and poultry applications require ton‐scale production of low‐cost, batch‐consistent phage products in an industry characterized by narrow profit margins. Manufacturing costs are primarily concentrated in two stages, upstream fermentation and downstream processing (Jurač et al. 2019; Mohammadi et al. 2025). Notably, downstream processing has received far less attention than upstream optimization and currently represents a principal bottleneck to commercialization (Tripathy et al. 2026). Moreover, parameters established at the laboratory scale do not always translate to industrial bioprocesses (Ali et al. 2019).

Upstream, batch fermentation remains the industrial norm due to its simplicity and high titers, yet it suffers from batch‐to‐batch variability. Continuous systems offer higher productivity, but prolonged host–phage co‐cultivation favors the emergence of phage‐resistant production strains; fed‐batch operation and two‐stage configurations that physically separate bacterial growth from phage infection can mitigate this risk (Nabergoj et al. 2018; Mutti and Corsini 2019). Production hosts must also be non‐toxigenic and free of prophages to prevent product contamination (Pirnay et al. 2015). Moreover, key infection parameters vary with bacterial growth rate and substrate availability, complicating process standardization (Nabergoj et al. 2018). Quality control further requires monitoring potency, genomic identity, purity, and pyrogenicity, while stabilization by drying extends shelf life at the cost of partial titer loss (Manohar and Ramesh 2019; Malik et al. 2023).

Crude phage lysates produced in Gram‐negative hosts contain bacterial LPS, host cell proteins, and nucleic acids. Because LPS is pyrogenic and tightly regulated, its removal is the central challenge of downstream processing (Szermer‐Olearnik and Boratyński 2015; Van Belleghem et al. 2017). While various purification strategies exist, ranging from filtration and organic solvent extraction to chromatographic workflows, they all involve trade‐offs between purity, recovery, and cost (Binte Mohamed Yakob Adil et al. 2024). Importantly, purification stringency should be fit for purpose, since endotoxin control is critical for parenteral products, whereas oral and feed applications deliver phages into an already LPS‐rich intestinal environment. Consequently, aligning purification depth with the administration route, rather than maximizing purity per se, is essential for containing production costs (Mutti and Corsini 2019).

Commercial feasibility is nonetheless demonstrated by existing precedents, as phage products are already marketed for food and agricultural applications (Vikram et al. 2021; Romero‐Calle et al. 2023), and phage cocktails have been deployed in field trials and on commercial poultry farms (Clavijo et al. 2022; Pino et al. 2025). Moreover, the self‐amplifying nature of phages implies that effective in vivo doses may be lower than those of comparable chemical additives, potentially reducing unit costs (Danis‐Wlodarczyk et al. 2021). However, systematic techno‐economic analyses across livestock species remain scarce, and fragmented regulations, including variable endotoxin limits and concentration caps, further complicate cost forecasting (Antoine et al. 2025; Mohammadi et al. 2025). Per‐species analyses are nevertheless beginning to emerge. A bioprocess model for a six‐phage cocktail against Salmonella in Colombian broilers estimated that a single 156‐L bioreactor coupled with 0.45/0.22‐μm filtration could supply 35% of the national broiler market (approximately 210 million birds) at a production cost of US$ 0.02 per chicken, with phage titer identified as the dominant cost driver rather than downstream recovery (Torres‐Acosta et al. 2019). Establishing standardized, low‐cost, and resistance‐aware manufacturing processes, together with quantitative cost–benefit evaluation, is therefore a prerequisite for industrial translation.

## 6 Conclusion

Against the backdrop of the escalating global antibiotic resistance crisis, phages have emerged as promising natural antimicrobial agents with high host specificity for intestinal health management in poultry and livestock. Current evidence demonstrates that phages can effectively target key pathogens including *Salmonella*, *Campylobacter*, pathogenic *Escherichia coli*, and *Clostridium perfringens*, exerting protective effects through pathogen elimination, microbiota modulation, intestinal barrier reinforcement, and immune response regulation. However, efficacy under field conditions is less predictable than controlled‐trial results suggest. Analyses of failed and non‐durable trials attribute this gap to identifiable causes, including insufficient delivery of active phages to the infection site, host immune clearance, inappropriate dosing relative to threshold pharmacology, and spatial exclusion coupled with ecological mismatch.

Translating phage technology into practical farming applications still requires addressing several critical challenges. The evolution of phage resistance in bacteria necessitates management through phage cocktail strategies and other approaches. Improving oral delivery efficiency depends on encapsulation technologies that balance gastrointestinal stability with production cost. Furthermore, the combined effects of phages with probiotics, plant extracts, and other non‐antibiotic interventions vary by combination, requiring systematic screening tailored to specific pathogen profiles and production contexts. Commercial translation further depends on large scale manufacturing in which downstream removal of endotoxins and residual bacterial proteins is achieved at a cost compatible with narrow livestock margins, with purification stringency matched to the intended route of administration.

Future research should quantify the cost‐benefit profiles of phage preparations across diverse livestock models and elucidate the biological mechanisms underlying phage‐mediated modulation of host gut microbiota to improve predictability. Long‐term ecological monitoring frameworks should be established to assess potential impacts on resistome dynamics and food safety. Through interdisciplinary collaboration and field validation, phage technology holds potential as an effective tool for maintaining intestinal health in the context of antibiotic reduction.

## Author Contributions

**Manman Song:** conceptualization, writing – original draft, writing – review and editing, visualization, formal analysis. **Yuying Fan:** writing – original draft, writing – review and editing, formal analysis. **Liguo Zhang:** writing – review and editing, supervision. **Wenhao Qi:** Formal analysis, Writing – review and editing, Visualization. **Lin Li:** writing – review and editing, formal analysis. **Gang Zhao:** conceptualization, supervision, writing – review and editing, project administration, funding acquisition, methodology. **Jianwei He:** conceptualization, methodology, writing – review and editing, supervision, project administration, funding acquisition.

## Ethics Statement

The authors have nothing to report.

## Conflicts of Interest

The authors declare no conflicts of interest.

## Data Availability

This is a literature review article. No new data were generated or analyzed in this study. All data discussed herein were derived from previously published studies, which are fully cited in the reference list.
